# Formulation of Self-Emulsifying Microemulsion for Acemetacin Using D-Optimal Design: Enteric-Coated Capsule for Targeted Intestinal Release and Bioavailability Enhancement

**DOI:** 10.3390/pharmaceutics17101270

**Published:** 2025-09-27

**Authors:** Zaineb Z. Abduljaleel, Khalid K. Al-Kinani

**Affiliations:** 1 Karbala Health Directorate, Ministry of Health, Karbala 56001, Iraq; 2 Department of Pharmaceutics, College of Pharmacy, University of Baghdad, Baghdad 10071, Iraq; khalidalkinani@copharm.uobaghdad.edu.iq

**Keywords:** acemetacin, self-emulsifying drug delivery systems, microemulsion, D-optimal design, antinociceptive activity, ex vivo permeation, capsule coating

## Abstract

**Objectives**: The current work aimed to formulate and optimize a self-emulsifying microemulsion drug delivery system (SEME) for acemetacin (ACM) to increase ACM’s aqueous solubility, improve oral bioavailability, and reduce gastrointestinal complications. **Methods**: Screening of components capable of enhancing ACM solubility was performed. Pseudo-ternary phase diagrams were performed to choose the optimal formulation ratio. The ACM-SEME formulation’s composition was optimized using D-optimal design. Oil, S_mix_, and water percentages were used as independent variables, while globule size, polydispersity index, ACM content, and in vitro ACM release after 90 min were used as dependent variables. Also, thermodynamic stability and transmittance percentage tests were studied. Zeta potential was assessed for the optimized ACM-SEME formulation, which was then subjected to spray drying. The dried ACM-SEME was characterized using field-emission scanning electron microscope, Fourier-transform infrared spectroscopy, X-ray diffraction, and differential scanning calorimetry. The dried ACM-SEME formulation was filled into hard gelatin capsules and coated with Eudragit L100 to achieve pH-dependent release. **Results**: The antinociceptive activity of ACM-SEME was evaluated in vivo using Eddy’s hot plate test in rats, revealing a significant prolongation of the noxious time threshold compared to control groups. Ex vivo permeation studies across rat intestinal tissue confirmed the enhanced permeation potential of the ACM-SEME. **Conclusions**: It was concluded that the developed ACM-SEME system demonstrated improved physicochemical properties, enhanced release behavior, and superior therapeutic performance, highlighting its potential as a safer and more effective oral delivery platform for ACM.

## 1. Introduction

Pain and inflammation are among the most common symptoms associated with both acute and chronic pathological conditions. Nonsteroidal anti-inflammatory drugs (NSAIDs) are widely used for the pharmacological management of these conditions due to their potent analgesic, anti-inflammatory, and antipyretic effects [1]. NSAIDs exert their therapeutic effects primarily through the inhibition of cyclooxygenase (COX) enzymes, which are involved in the biosynthesis of prostaglandins, a key mediator of inflammation and pain. Despite their efficacy, the long-term and high-dose use of conventional NSAIDs is frequently associated with significant adverse effects, particularly on the gastrointestinal tract, including gastric irritation, ulceration, and bleeding [2]. These drawbacks have necessitated the exploration of alternative anti-inflammatory agents that combine improved safety profiles with effective symptom relief.

Acemetacin (ACM) is a promising candidate in this regard. It is a glycolic acid ester prodrug of indomethacin, developed with the aim of reducing the gastrointestinal toxicity associated with its parent compound. Following oral administration, ACM undergoes enzymatic hydrolysis in the intestinal wall and plasma, liberating the active parent compound “indomethacin”. This prodrug design significantly reduces direct gastric irritation compared with indomethacin, thereby offering an inherent pharmacological safety advantage [3]. While indomethacin can be delivered as an enteric-coated capsule to reduce gastric exposure, premature or incomplete coating protection may still result in mucosal injury. It has been reported that ACM used to maintain equivalent analgesic and anti-inflammatory efficacy with improved tolerability compared to indomethacin. However, clinical reports have shown that, although ACM is generally better tolerated than indomethacin, residual gastric side effects can still occur, particularly during long-term therapy or in sensitive patients [4].

The clinical utility of ACM is constrained by its poor aqueous solubility (practically insoluble), which limits its dissolution rate and oral bioavailability. The oral bioavailability of ACM has been reported at 64–66% following a single dose in humans; ACM is commonly administered at 60–180 mg/day in divided doses [5]. According to the Biopharmaceutics Classification System (BCS), ACM falls under Class II, characterized by high membrane permeability but low water solubility [6]. The physicochemical profile of ACM includes a log *p* value of approximately 4.2, indicating significant lipophilicity, and a pKa of 2.6. Its aqueous solubility is extremely limited, whereas it demonstrates solubility in various organic solvents such as ethanol, acetone, and dimethyl sulfoxide (DMSO) [7].

Several improved drug delivery techniques have been studied to overcome the solubility and bioavailability challenges associated with ACM. Self-micro-emulsifying drug delivery systems (SEME) are one of the most popular. SEME comprises oil, surfactants, and co-surfactants that spontaneously form fine oil-in-water microemulsions when exposed to the aqueous environment of the gastrointestinal system under gentle agitation [8]. The spontaneous emulsification process generates micro- or nano-sized droplets, thereby increasing the interfacial surface area available for drug release and absorption. This, in turn, facilitates improved drug solubilization and significantly enhances the rate and extent of drug absorption across the gastrointestinal epithelium.

SEME formulation performance is impacted by a variety of factors, including oil type and concentration, surfactant and co-surfactant selection, and drug-to-excipient ratios. Oils are not simply drug solubilizers; they also promote lymphatic absorption of lipophilic medicines [9]. Surfactants reduce interfacial tension and stabilize microemulsions, whereas co-surfactants increase the flexibility of the interfacial layer, allowing small droplets to form spontaneously when diluted [10]. Following oral administration, SEME rapidly disperses in GI fluids, generating a thermodynamically stable microemulsion that keeps the medication solubilized, preventing precipitation and encouraging continuous absorption throughout the gastrointestinal system.

While liquid SEME formulations are successful at increasing bioavailability, they may have drawbacks in terms of physical stability, handling, and patient compliance. As a result, turning SEME into a solid dosage form, such as via adsorption onto solid carriers or spray-drying, is a typical technique for overcoming these restrictions [11]. Furthermore, the solidified SEME formulation can be encapsulated within enteric-coated capsules to preserve the acid-labile ACM from breakdown in the stomach and reduce residual GI discomfort. These capsules are engineered to withstand the acidic gastric environment and break-down only at higher pH levels in the intestine, resulting in targeted medication release in the small intestine’s neutral to alkaline environment. This method is especially useful for medicines like ACM, which are both poorly soluble and possibly irritating to GIT.

The major goal of the current work was to design and optimize ACM-SEME in order to improve its solubility and oral bioavailability. The refined ACM-SEME formulation was then solidified and encased in enteric-coated capsules to prevent gastrointestinal adverse effects and ensure site-specific drug delivery. The dual protective strategy, which combines pharmacological protection at the molecular level (ACM is a prodrug) and formulation-based protection at the dosage form level (via enteric coating), represents a synergistic approach to maximizing gastrointestinal tolerability while maintaining therapeutic efficacy. The antinociceptive efficacy of the final formulation was tested in rats using Eddy’s hot plate method, revealing its potential as a safer and more effective alternative to traditional NSAID therapy.

## 2. Materials and Methods

### 2.1. Materials

Acemetacin was obtained from Bide Pharmatech Ltd., Shanghai, China. Imwitor 308 was supplied by IOI Oleochemical GmbH, Germany. Tween 20 was supplied by Riedel-De-Haen, Germany. Transcutol P was provided by Gattefossé, Lyon, France. Polyethylene glycol (PEG 400) was supplied by Fluka Chemi AG, Switzerland. Acetone was purchased from Loba Chemie, India, while buffer reagents were supplied by Sigma-Aldrich, St. Louis, MO, USA. Aerosil, used as a solid carrier, was obtained from Evonik Industries AG, Germany. All chemicals and reagents were of analytical grade and used as received.

### 2.2. Screening of Components for ACM-SEME

The solubility of ACM in different oils, surfactants, and cosurfactants was studied to screen the components that can be utilized in the preparation of ACM-SEME. To determine the solubility of ACM, 2 mL of each vehicle (oils, surfactants, and cosurfactants) was dispensed into borosilicate glass test tubes, followed by the addition of an excess of ACM. The supersaturated solutions were stirred and incubated in water bath shaker at 37 °C for 72 h, with oscillations at a frequency of 100 per minute to guarantee complete mixing and dissolution. After achieving equilibrium, the samples were centrifuged at 4000× *g* for 25 min to remove undissolved solids [12]. The filtrates were subsequently diluted with ethanol, and the concentration of ACM in each sample was determined using UV spectrophotometric analysis. All spectrophotometric measurements were carried out using a Shimadzu UV-1900 spectrophotometer equipped with LabSolutions version 5.3 software (Shimadzu, Japan). The wavelength of maximum absorption (λmax) for ACM was determined to be 318 nm. Calibration was performed over six concentrations (5, 15, 25, 35, 45, and 55 µg/mL). The calibration curve for ACM was constructed by plotting absorbance against concentration, yielding the regression equation *y* = 0.0152 *x* − 0.0095 with a regression coefficient (R^2^ = 0.9996).

### 2.3. Construction of Pseudo-Ternary Phase Diagram

Imwitor 308, Tween 20, and Transcutol-P were chosen as the oil phase, surfactant, and co-surfactant, respectively, since they had the highest solubility of ACM in different components. Tween 20 and Transcutol-P have been designed in various ratios, including 1:1, 1:2, 2:1, 3:1, 1:3, 2:3, 3:2, and 1:4. The surfactant-co-surfactant mixture is known as Smix. Various Smix-to-oil ratios were then developed, including 1:9, 2:8, 3:7, 4:6, 5:5, 6:4, 7:3, 8:2, and 9:1. Water was added dropwise to each mixture until the first sign of turbidity, indicating the phase boundary. The pseudo-ternary diagram was plotted using ProSim version 1.0.3 software (ProSim Ternary Diagram, ProSim, Cedex, France) [13].

### 2.4. Experimental Design for Optimization of Liquid ACM-SEME

D-optimal design was constructed using design of experiment Design Expert^®^ (Version 13.0.0, Stat-Ease Inc., Minneapolis, MN, USA). The D-optimal design was constructed to investigate the effects of the independent variables (Imwitor 308 oil %, Smix % (Tween 20 and Transcutol-P in a 3:1 ratio), and water %) on the dependent variables: globule size (PS), polydispersity index (PDI), ACM content, and cumulative percentage of ACM released after 90 min. The selection of formulation percentages was guided by the results of a previously prepared pseudo-ternary phase diagram. The sum of all components in a formulation always equals 100 percent [14]. The software generated sixteen experimental formulations (11 formulations and 5 overall center point replicates), as presented in Table 1.

Various mathematical models, including linear, quadratic, special cubic, cubic, special quartic, and quartic models, were evaluated using the software to determine the most suitable model for the mixture D-optimal design. The significance of each model was assessed by comparing statistical parameters such as *p*-value, F-value, coefficient of determination (R^2^), adjusted R^2^, predicted R^2^, and Adeq Precision. To confirm the selected model validity, the optimal model was chosen based on the most significant *p*-value and the highest values of adjusted R^2^ and predicted R^2^, with a difference of no more than 0.2. Additionally, the model validity was further confirmed by measuring Adeq Precision, which measures the signal-to-noise ratio [12]. The optimal formulation was selected based on the smallest PS, the lowest PDI, high ACM content, and the highest cumulative percentage drug release after 90 min.

### 2.5. Preparation of Acemetacin Self-Micro-Emulsifying Drug Delivery Systems

The ACM–SEME formulations described in Table 1 were prepared by combining Imwitor 308 oil with a Smix composed of Tween 20 and Transcutol-P in a 3:1 ratio, followed by magnetic stirring until a homogeneous mixture was obtained. Subsequently, 30 mg of ACM was incorporated and stirred until completely dissolved. Water was then added gradually, dropwise, with continuous stirring at room temperature to yield a transparent and isotropic microemulsion [12,15].

### 2.6. Characterization of the Prepared Liquid ACM-SEME

#### 2.6.1. Thermodynamic Stability Study

To evaluate the thermodynamic stability of the prepared formulation, a series of stress tests were conducted to simulate potential physical instabilities under various environmental conditions. These tests aimed to assess the formulation’s ability to withstand temperature fluctuations and mechanical stress without exhibiting signs of phase separation, creaming, or precipitation. The study included three main tests: the Centrifugation Test, the Heating–Cooling Cycle, and the Freezing–Thawing Test. Each test was designed to challenge the formulation under accelerated conditions to predict its long-term stability behavior [13].

##### Centrifugation Test

In the Centrifugation Test, all liquid ACM-SEME formulations were exposed to centrifugation at 4000 rpm for 30 min. Formulations that showed no signs of phase separation, creaming, or cracking were considered physically stable.

##### Heating-Cooling Cycle

Six alternating cycles between 4 ± 2 °C and 40 ± 2 °C were performed on all liquid ACM-SEME formulations, with a minimum of 48 h of storage at each temperature.

##### Freezing–Thawing Test

Three freeze–thaw cycles between −20 ± 2 °C and 25 ± 2 °C were performed on all liquid ACM-SEME formulations, with at least 48 h of incubation at each temperature.

#### 2.6.2. Globule Size and Polydispersity Index Determination

The globule size and PDI of the liquid ACM-SEME were assessed by diluting the formulation tenfold with deionized water and measuring it using a Malvern Zetasizer Nano ZS equipped with a clear disposable zeta cell (Model Nano ZS, Malvern Instruments, Malvern, UK) [16].

#### 2.6.3. Determination of ACM Content in Liquid ACM-SEME

An aliquot of 0.1 mL from each SEME formulation was transferred into a 10 mL volumetric flask and diluted to volume with ethanol. The resulting mixture was sonicated for 60 min to ensure complete dispersion and solubilization of the active component. Following sonication, the solutions were filtered using a 0.45 µm syringe filter to remove any particulate matter. The clear filtrates were then subjected to UV-Vis spectrophotometric analysis to quantify the ACM content, using a previously constructed calibration curve prepared in ethanol [17].

#### 2.6.4. In Vitro Release of Liquid ACM-SEME

The in vitro release of the ACM was evaluated using the dialysis bag method with a USP type II dissolution apparatus. Briefly, a dialysis bag with a molecular weight cut-off of 12 kDa was soaked in phosphate buffer (6.8) overnight to open its pores and remove any preservative materials; then, from each SEME formulation (Table 1), an equivalent amount corresponding to 15 mg of ACM was placed into the dialysis bag and compared with an ACM suspension containing the same drug amount. The membrane was securely sealed and immersed in 500 mL of phosphate buffer (pH 6.8). The release study was conducted at a constant temperature of 37 ± 0.5 °C with a stirring speed of 100 rpm. At predetermined time intervals (5, 15, 30, 45, 60, and 90 min), 5 mL samples were withdrawn from the dissolution medium and immediately replaced with an equal volume of fresh buffer to maintain sink conditions. The collected samples were analyzed using a UV spectrophotometer [12].

#### 2.6.5. Percentage Transmittance (T%)

A Shimadzu UV/VIS (UV-1900 Pharma Spec, Shimadzu, Japan) spectrophotometer was used to calculate the percentage transmittance. Using deionized water, one milliliter of the liquid ACM-SEME formulation was diluted 100 times before being examined at 650 nm with deionized water as the reference material [18].

#### 2.6.6. Zeta Potential of the Optimized Liquid ACM-SEME Formulation

The zeta potential of the optimized formulation was assessed by diluting the formulation tenfold with distilled water and measuring it using a Malvern Zetasizer Nano ZS equipped with a clear disposable zeta cell (Model Nano ZS, Malvern Instruments, Malvern, UK) [19].

#### 2.6.7. Drying of Liquid ACM-SEME Optimized Formulation

Solid ACM-SEME was formulated using a spray-drying method employing a Büchi B290 mini spray-dryer (Büchi Labortechnik AG, Flawil, Switzerland). Aerosil 200 was selected as the solid carrier due to its high specific surface area, excellent flowability, and superior adsorption capacity. The liquid ACM-SEME optimized formulation (F5) was mixed with Aerosil 200 at a ratio of 1:0.5, and then 200 mL of deionized water was added under continuous stirring. The resultant dispersion was spray-dried through a 0.07 mm nozzle. The process parameters were optimized with an inlet temperature of 120 °C, an outlet temperature of 70 °C, a feed rate of 5 mL/min, and an aspiration rate set at 85% [20]. The spray-dried powder demonstrated a drug loading of approximately 8% *w*/*w*, calculated on the basis of ACM content relative to total powder mass.

#### 2.6.8. Characterization of the Dried ACM-SEME Optimized Formulation

##### Morphological Analysis of Dried ACM-SEME Optimized Formulation

Field emission scanning electron microscopy (FE-SEM) is employed to visualize the ACM-SEME. In this study, the surface morphology of the spray dried ACM-SEME was investigated using an FE-SEM instrument (Inspect F50, FEI Company). Samples were mounted on specimen holders using double-sided carbon adhesive tape. Prior to imaging, a uniform gold coating was applied via sputter coating using an automated fine coater to enhance surface conductivity and image clarity. The analysis was performed under varying magnification levels to obtain detailed structural insights [21].

##### FTIR Determination

FTIR analysis was conducted to assess compatibility and detect any potential intermolecular interactions, whether physical or chemical, between the ACM and the other formulation ingredients. The FTIR spectrum of the spray-dried optimized formulation was compared with the spectra of its individual components using FTIR (8100M, Shimadzu, Kyoto, Japan). Samples (1–2 mg) were ground, mixed with potassium bromide, and compressed into disks, and FTIR spectra were recorded. The spectroscopic analysis was carried out within the range of 4000–400 cm^−1^ [22].

##### X-Ray Diffraction (XRD)

The PANalytical Aeris with PIXcel1DMedipix3 detector and CuKα radiation source was utilized to identify the nature of structures of both ACM and dried ACM-SEME optimized formulation. The scanning was conducted in the Theta-2 axis mode within a range of 5–100° [23].

##### Differential Scanning Calorimetry

DSC analysis of both ACM and dried ACM-SEME was carried out using a Shimadzu DSC-60 (Japan). Samples (2–4 mg) were accurately weighed and placed in an aluminum pan and heated at the rate of 10 °C/min to a temperature of 200 °C. The instrument was calibrated with indium, and nitrogen was used as purge gas through DSC cells with a flow rate of 30 mL/min.

##### Capsule Filling and Coating

Empty hard gelatin capsules were manually filled with spray-dried ACM-SEME, with each capsule containing 375 ± 5 mg of powder corresponding to 30 mg of ACM. Following filling, the capsules were subjected to a coating process using Eudragit^®^ L100 dispersion to achieve delayed-release properties. The Eudragit^®^ L100 dispersion was prepared by dissolving 1 gm of Eudragit L 100 in 10 mL of acetone and 0.2 mL of PEG, followed by stirring for 15 min. Subsequently, the coating was applied by dipping the filled capsules three times into the dispersion, with each dipping followed by drying at using hot air (45 °C) temperature to ensure proper film formation. The coating process resulted in a weight gain of 8–10% *w*/*w* [24,25].

##### In Vitro Release Study of Eudragit L100-Coated Capsules Filled with Spray-Dried ACM-SEME

The release study was carried out in a USP dissolution apparatus II by changing the pH method. The capsules were transferred to the dissolution medium, and samples were taken at selected time intervals, filtered through syringe filter, and analyzed by UV spectrophotometer of Shimadzu-1900 with LabSolutions version 5.3 software (Shimadzu, Japan) at 318 nm. The continuous dissolution method USP was used by simulating conditions of the GI tract. In this study capsules were added to 250 mL of 0.1 N HCl (pH 1.2) for 2 h. At the end of 2 h, 233.3 mL of 0.2 M tribasic sodium phosphate was added to all the dissolution vessels, and the pH was adjusted to 6.8 using 2 M NaOH [26].

##### Effect of ACM on the Noxious Time Threshold on Rats

The antinociceptive activity of the formulations was evaluated using the Eddy’s hot plate method. Male Wistar rats (180–200 g) were divided into four groups (*n* = 6 each): (i) negative control (no treatment), (ii) placebo (blank SEME formulation), (iii) positive control (ACM suspension, equivalent to 5 mg/kg), and (iv) test group (ACM-SEME, equivalent to 5 mg/kg). Thirty minutes after oral administration, each rat was individually placed on a hot plate maintained at 50 ± 0.5 °C, and the latency time until the first nociceptive response (hind paw licking, withdrawal, or jumping) was recorded. A cut-off time of 30 s was applied to prevent tissue injury. Increased reaction latency compared with the negative and placebo groups was considered evidence of analgesic efficacy [4,27]. The study was conducted at the College of Pharmacy, University of Baghdad, and received ethical approval from the college’s Research Ethics Committee (Approval number: REC032440R). All animals were handled in accordance with the guidelines for the care and use of laboratory animals, as outlined by the US National Institutes of Health (NIH Publication No. 85-23, revised 1996). Before the study began, the animals were fasted from food but had unlimited access to water for the whole night.

##### Ex Vivo Permeation Study

The Ex Vivo Permeation Study was performed using the non-everted intestinal sac method. Before the study began, the animals were fasted from food but had unlimited access to water for the whole night. Anesthesia was induced using diethyl ether inhalation. A midline abdominal incision (3–4 cm) was performed to expose the small intestine, which was then isolated. A clean 10 cm segment of the duodenum was excised, rinsed, and placed in fresh phosphate-buffered saline pH 7.4 maintained at 37 °C after removing the associated mesentery. The intestinal segments were then flushed with 0.9% sodium chloride solution using a 5 mL syringe to clear any residual contents [28].

Using a blunt-end needle, each non-everted intestinal sac (10 cm length) was filled with 1 mL of the reconstituted (with distilled water) spray-dried ACM-SEME (equivalent to 15 mg ACM) and securely tied at the opposite end to form a sealed sac. A similar procedure was applied for the ACM suspension, which was prepared by adding 15 mg ACM in 1 mL of distilled water. Both sacs were immediately suspended from the paddle of a USP dissolution apparatus (Type II) containing 500 mL of PBS (pH 7.4). The permeation medium was continuously gassed with oxygen (20 bubbles/minute). Samples (5 mL) were collected at predetermined intervals (5, 10, 15, 30, 45, 60, 90, and 120 min) and were immediately replaced with an equal volume of fresh phosphate-buffered saline pH 7.4. Drug concentrations in the withdrawn samples were determined using a UV spectrophotometer at a wavelength of 318 nm, based on a previously established calibration curve for ACM in PBS. All experiments were conducted in triplicate, and results were expressed as mean ± standard deviation [29].

## 3. Results and Discussion

### 3.1. Screening and Selection of ACM-SEME Components

The selection of suitable components is a critical factor in the development of clear, homogeneous, and stable microemulsions. Solubility plays a pivotal role in the screening process, as it directly influences the solubilization efficiency of poorly water-soluble drugs like ACM in SEME, which is essential to ensure the optimal performance and stability of the final ACM-SEME formulation. In solubility studies of poorly water-soluble drugs, prolonged equilibration times are often required to ensure attainment of true saturation. In our work, an incubation period of 72 h was adopted after preliminary checks at 48 h indicated that the solubility values had not fully stabilized, whereas no further increase was observed between 72 and 96 h, confirming equilibrium. The variation in solubility values between 48 h and 72 h remained within 5%, supporting the selection of 72 h as the optimal equilibration time. Similar findings have been reported for other poorly soluble compounds, where equilibration periods of 48–72 h were required to achieve stable solubility values, emphasizing the necessity of extended incubation for accurate determination [30,31,32]

**Figure 1:**
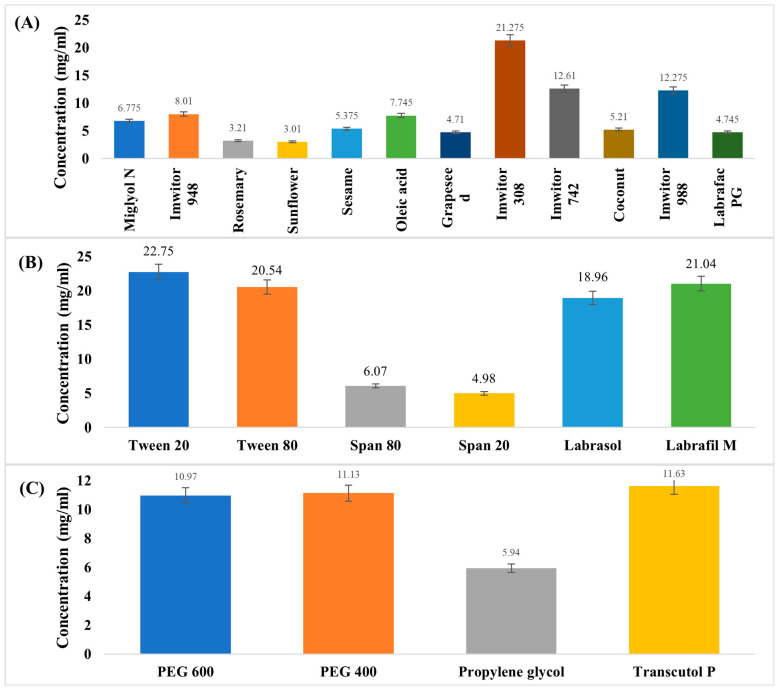
The saturated solubility of ACM in (**A**) different oils, (**B**) different surfactants, and (**C**) different co-surfactants.

The solubility of ACM in different components is illustrated in Figure 1. It was observed that ACM has the highest solubility in Imwitor 308 oil, Tween surfactant, and Transcutol-P as co-surfactant. Imwitor 308 primarily consists of glyceryl monocaprylate monoester (80%), commonly employed as a solubilizer, penetration enhancer, plasticizer, and bioavailability enhancer by facilitating drug permeability and solubilization [33]. Tween 20 and Transcutol-P are known for their high hydrophilic-lipophilic balance values, indicating their appropriateness for stable SEME formation. Furthermore, their low oral toxicity profile is an important consideration in ensuring the safety of the delivery system [34].

### 3.2. Pseudo-Ternary Phase Diagram and Microemulsion Region

**Figure 2:**
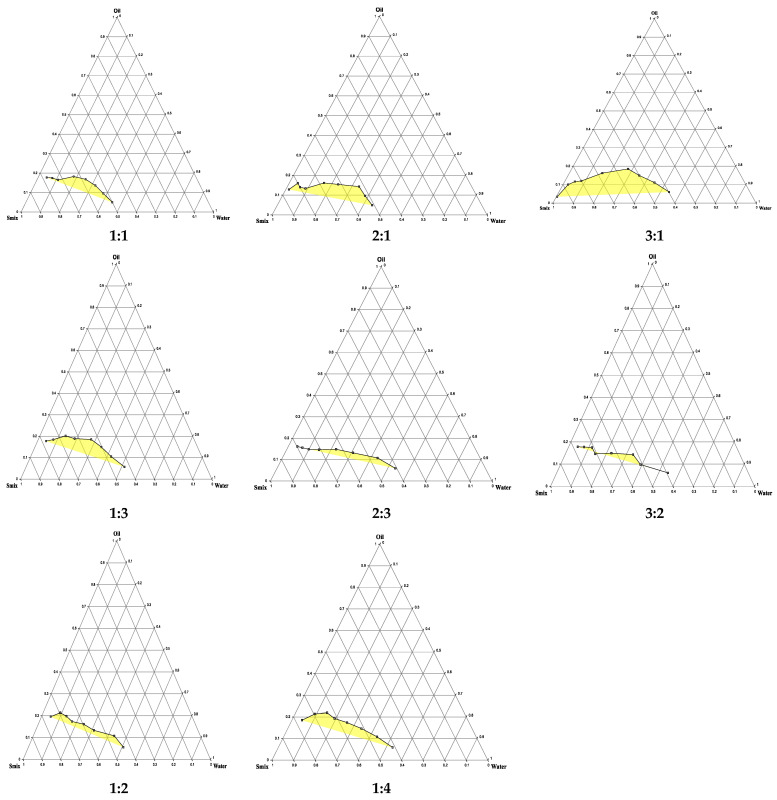
The pseudo-ternary phase diagrams for oil (Imwitor 308), Smix (Tween: Transcutol-P) at different ratios (1:1, 2:1, 3:1, 1:3, 2:3, 3:2,1:2 and 1:4), and water. The yellow-highlighted region represents the one-phase region, whereas the uncolored area corresponds to the two-phase microemulsion region.

The pseudo-ternary phase diagrams (Figure 2) illustrate the impact of oil, Smix, and water quantities on the formation of microemulsion zones. The variation in microemulsion area across different Smix ratios demonstrated the importance of surfactant-to-cosurfactant balance in optimizing the emulsification process. At lower Smix ratios (1:1), emulsification capacity is low, most likely due to interfacial tension. As the surfactant content increases (2:1 and 3:1), the interfacial tension between oil and water is reduced more efficiently, facilitating the spontaneous generation of microemulsions and widening the self-emulsifying zone. The 3:1 Smix ratio resulted in the largest microemulsion area, demonstrating an ideal balance for stabilizing oil droplets and increasing water solubility. This is due to the surfactant’s dominance, which enhances interfacial stability and droplet dispersion [35]. However, increasing the co-surfactant percentage (1:3 and 1:4) disrupted this balance, most likely leading to excessive fluidization of the interfacial film or dilution of the surfactant action, resulting in a smaller self-emulsification zone. Excess co-surfactants can also increase system polarity, limiting its capacity to dissolve hydrophobic oil components [36]. The diagrams also showed that a moderate oil percentage (10–20% *w*/*w* oil), when combined with a high Smix percentage (40–60% *w*/*w* Smix), promotes microemulsion production, while at a high oil percentage, Smix’s emulsification ability is overwhelmed, limiting the creation of stable microemulsions [37]. Finally, the findings show that the Smix ratio is an important aspect in defining the microemulsion region, and the 3:1 ratio provides the best ratio for formulating a stable and efficient SEME, so it was selected to be involved in the experimental design.

### 3.3. Thermodynamic Stability of Liquid ACM-SEME

All ACM-SEME formulations withstand centrifugation, temperature cycling, and freeze–thaw stress indicates excellent thermodynamic stability. The absence of physical changes under these stress conditions suggests strong resistance to mechanical and thermal destabilization forces. This behavior can be attributed to the formulation components, particularly the surfactant and co-surfactant system, which likely enhanced interfacial stability and prevented coalescence or phase separation. Such stability under accelerated conditions is a strong indicator of long-term storage stability.

### 3.4. Experimental Design for Optimization of Liquid ACM-SEME

In mixture experiments, the component levels cannot be varied independently or assigned randomly, as their total must always sum to 100%. Conventional experimental designs do not account for this constraint, which may compromise their ability to accurately predict outcomes [38]. In such cases, a D-optimal design is preferable, as it maximizes prediction accuracy by selecting the most relevant experimental runs while minimizing variability in projected model coefficients [39]. The D-optimal design was constructed to investigate the effects of the independent variables (oil % (10–25%), S_mix_ % (55–65%), and water % (10–35%)) on the dependent variables: PS, PDI, ACM content, and cumulative percentage of ACM released after 90 min.

The results of PS, PDI, ACM content, and % ACM released after 90 min are illustrated in Table 1.

**Figure 3:**
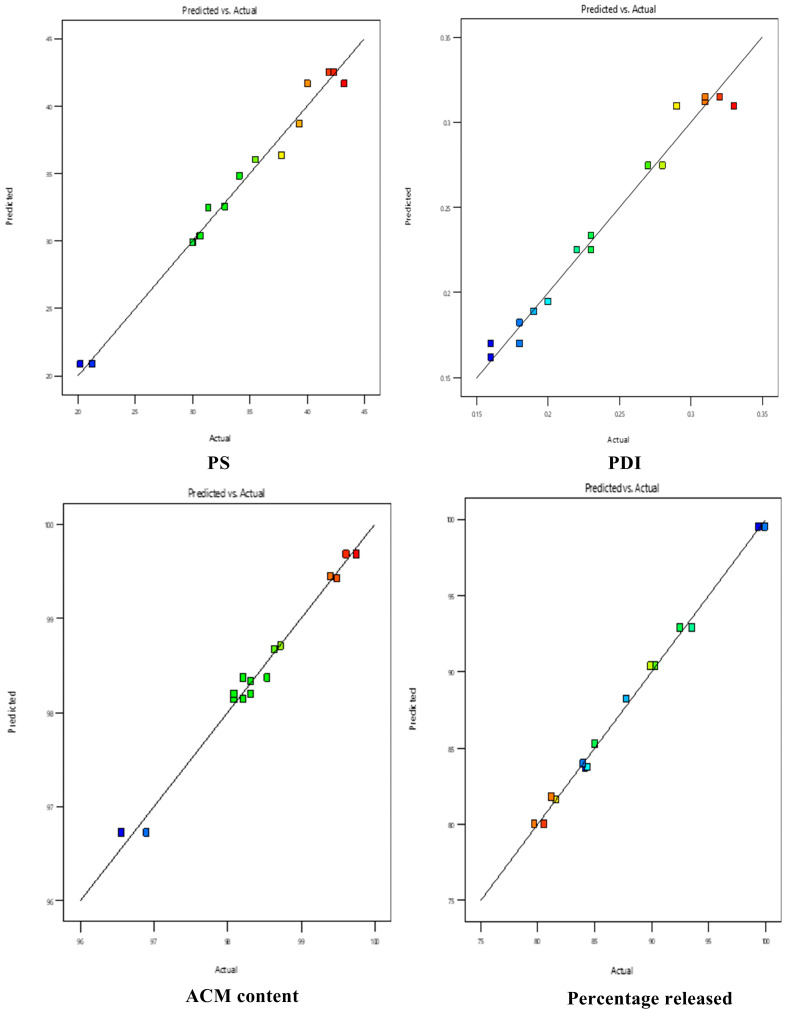
Predicted vs. actual for PS (globule size), PDI (polydispersity index), ACM content, and percentage of drug released. Blue indicates lower values, green indicates moderate values, and yellow/orange to red indicates higher values.

Using experimental design, it was observed that the best models for PS, PDI, ACM content, and % ACM released after 90 min were Special Quartic, cubic, cubic, and quadratic, respectively. According to fit statistics for the dependent variables illustrated in Table 2, these models showed reasonable agreement between the predicted R^2^ value and the adjusted R^2^, with a difference of less than 0.2. Moreover, the adequate precision value was above the threshold of 4, indicating a strong signal-to-noise ratio and confirming the suitability of the model to explore the design space effectively. Furthermore, the close alignment of predicted versus actual values further validated the robustness of the models, as most data points clustered near the ideal fit line (Figure 3).

**Figure 4:**
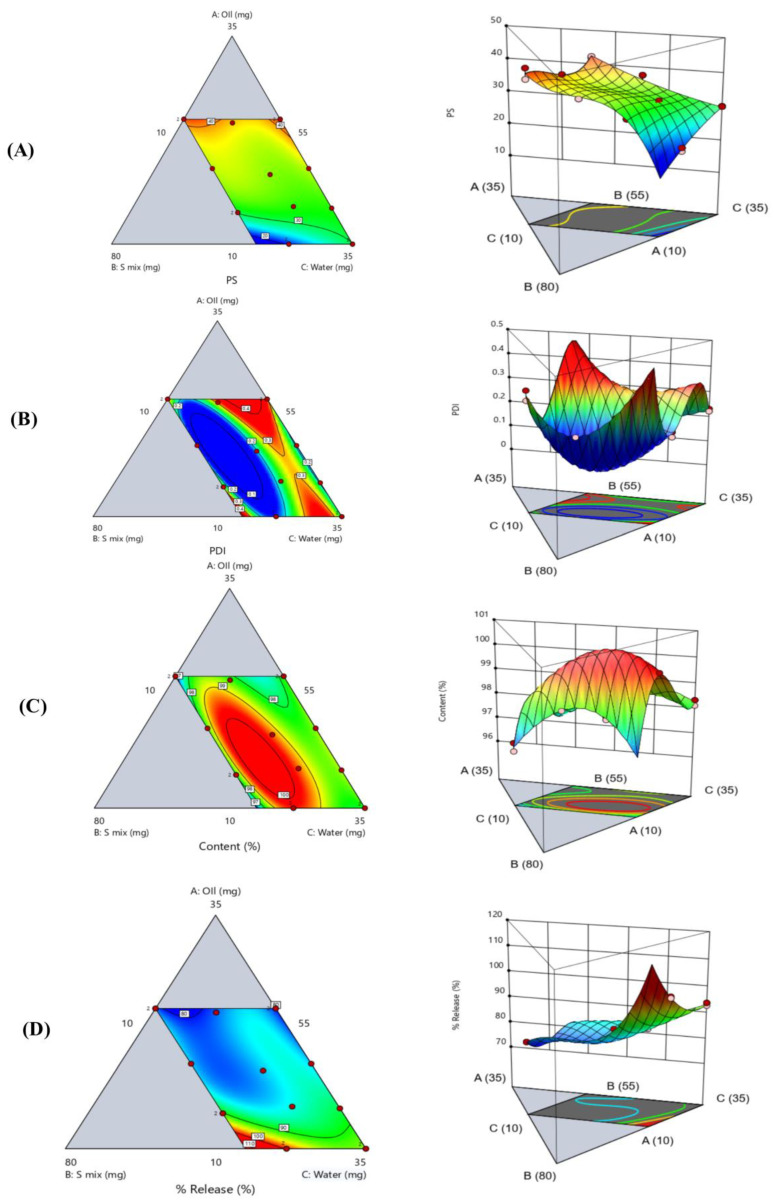
The contour and 3D surface plots for (**A**) (globule size), (**B**) (polydispersity index), (**C**) (ACM content), and (**D**) (percentage of drug released). Blue indicates lower values, green moderate values, and red/yellow higher values.

The coded equation for each response (Table 2), ternary contour plot and 3D response surface plot (Figure 4) together provide a comprehensive visualization of how oil, S_mix_, and water percentages affect dependent variables.

### 3.5. Globule Size and Polydispersity Index

The PS of liquid ACM-SEME is regarded as a significant component, influencing overall performance because it has a direct impact on the interfacial area. This, in turn, affects the drug’s release and absorption characteristics (both rate and extent) and, consequently, its bioavailability [40].

The PS polynomial equation showed that oil percentage showed a strong positive influence on PS (+56.42), indicating that increasing oil content tends to enlarge PS. This can be attributed to the fact that oil-rich systems typically have a higher viscosity and greater internal phase volume, both of which hinder efficient droplet breakup during emulsification, resulting in larger particles [41,42]. Conversely, the S_mix_ percentage has a strong negative effect (–31.41), suggesting that increasing the S_mix_ significantly reduces PS. This behavior is well-documented [43], as surfactants lower the interfacial tension between oil and water, promoting the formation of smaller, more stable droplets due to efficient interfacial tension reduction [44,45]. The water percentage also exerts a positive effect (+29.87) on PS, though to a lesser extent than oil. The ternary contour plot and 3D surface plot (Figure 4A) showed that where water content is high and S_mix_ is moderate to low, smaller PS (20–30 nm) is obtained. This may be attributed to the dilution effect, where higher water content facilitates better dispersion of the oil phase and more efficient emulsification, provided that the S_mix_ content remains within an optimal range. Adequate hydration supports the formation of fine droplets, reducing the chances of droplet coalescence [46]. Furthermore, the ternary contour plot and 3D surface plot (Figure 4A) demonstrated that while S_mix_ helps reduce interfacial tension, excessive concentration without adequate water or oil balance may lead to increased droplet aggregation or coalescence, ultimately increasing PS due to decreased efficiency of emulsification. This outcome aligns with known emulsification mechanisms, where the absence of sufficient surfactant coverage results in interfacial instability and increased droplet size [47]. Additionally, the middle region of the plot, where oil, S_mix_, and water are relatively balanced, shows intermediate particle sizes (30–35 nm). This implies that particle size is controlled by the interaction of all three factors rather than being exclusively dependent on one.

Droplet size distribution is a critical parameter in SEME formulation because it affects system uniformity, stability, and performance. The PDI quantifies this distribution by comparing the standard deviation with the mean droplet size. PDI measures the homogeneity of droplets inside a microemulsion, indicating the overall quality of the dispersion. Lower PDI values, particularly those near zero, suggest a narrow size distribution, implying a more homogeneous and monodispersed system. Such homogeneity is required to ensure constant medication release, improved absorption, and formulation stability over time. Importantly, PDI is inversely related to the stability and homogeneity of droplet size in the SEME; greater values indicate heterogeneity and probable instability [48].

The PDI equation (Table 2), contour and 3D surface plots (Figure 4B) showed that the PDI values were highly dependent on the proportions of oil, S_mix_, and water within the formulation, if an imbalance either due to excessive oil that surpasses the emulsifying capacity of S_mix_, or excessive water that dilutes the surfactant system leads to poor droplet stabilization and greater size variability [49]. The 3D surface plot further supports these findings by revealing a pronounced concave region at the center of the design space, representing the optimal PDI zone. This concavity highlights the precise formulation window where oil, S_mix_, and water interact synergistically to yield efficient emulsification and minimal droplet polydispersity, a hallmark of robust and stable SEME. It is also observed that when the oil percentage increases, so does the PDI, which might be explained as follows: when the oil percentage increases, the volume of the dispersed phase increases, necessitating a higher amount of surfactants to effectively stabilize the increased number of droplets produced [50]. If the S_mix_ percentage is not increased correspondingly, certain droplets will remain insufficiently covered, resulting in instability and coalescence. Furthermore, increasing oil percentage raises the viscosity of the internal phase, which may limit efficient emulsification and promote the formation of droplets of different sizes, resulting in a broader size distribution [51]. The effect of S_mix_ percentage on PS and PDI is complex, as it is influenced by the oil-S_mix_ ratio. While moderate quantities of S_mix_ help to reduce PS and improve PDI, overly high percentages can have the reverse effect. At high S_mix_ levels, the system may become excessively fluid or thermodynamically unstable. This instability might cause excessive solubilization of the oil phase or the development of irregular and non-uniform droplets during emulsion. Such inconsistencies lead to an increase in the PDI [52,53].

### 3.6. ACM Content in Liquid ACM-SEME

ACM content results were found to be within the recognized USP requirement range of 85% to 115% [54]. The drug content of the prepared formulations ranged from 96.56% to 99.75%, reflecting a high degree of uniformity and successful incorporation of the ACM across all experimental runs without precipitation or degradation, revealing homogeneity and stability of the prepared formulation. The model equation coefficients (Table 2) show that oil concentration has a positive effect on ACM content, as demonstrated by their positive coefficients (+99.36). This behavior is well-supported in literature and is primarily related to the greater solubilization ability of the oil phase. Increasing the oil concentration enlarges the lipophilic domain of the formulation, which can accommodate a bigger amount of the ACM [52,55,56]. In contrast, Smix concentration exhibits a large negative main effect (−86.55), showing that an excessive amount of Smix may lead to a drop in drug content, either due to saturation of the surfactant system or destabilization of the microemulsion structure at higher concentrations [57].

The ternary contour plot and 3D response surface plot (Figure 4C) together showed a color gradient from blue (lowest ACM content, 96.56%) to red (highest, 99.75%), highlighting that formulations with moderate oil, high S_mix_, and sufficient water yield the best drug content. The highest values appear near the center of the S_mix_–water axis, indicating a strong positive interaction between these two components. In contrast, formulations with high oil or low S_mix_/water show reduced ACM content due to poor emulsification [13].

### 3.7. In Vitro Release of Liquid ACM-SEME

**Figure 5:**
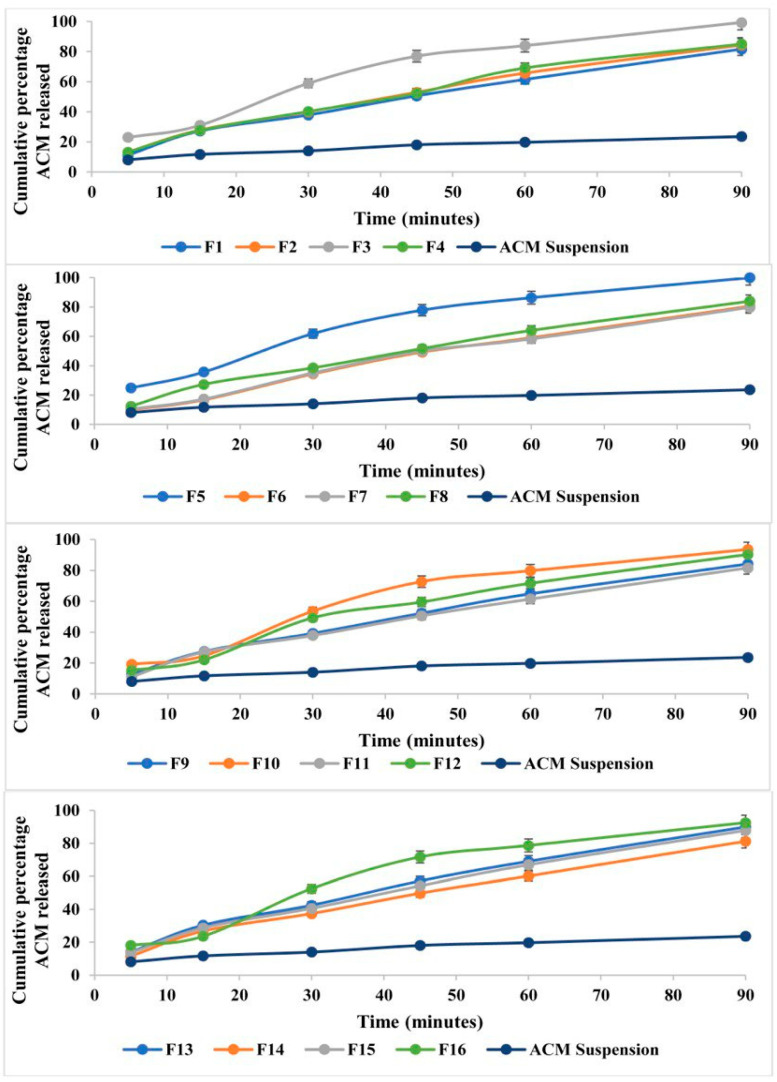
In vitro release profiles of ACM-SEME formulations compared with ACM suspension in phosphate buffer (pH 6.8, *n* = 3, mean ± SD).

The in vitro releases of the sixteen ACM-loaded formulations (Figure 5) were evaluated at multiple time intervals (5, 15, 30, 45, 60, and 90 min). All formulations exhibited a time-dependent release pattern, with the cumulative percentage of ACM released increasing progressively over time.

During the first 5 min, the ACM release ranged from 9.99% for formulation F6 to 24.87% for formulation F5, indicating a rapid initial burst in certain formulations. By 15 min, the release values had extended between 16.79% (F6) and 35.71% (F5). At 30 min, distinct differences among formulations became apparent, with F3, F5, F10, and F16 exceeding 50% release. Notably, F5 demonstrated the highest release at this point (61.86%), while F6 remained the lowest (34.44%).

By 60 min, most formulations had released more than 60% of the drug, with F5 maintaining the highest release (~86.31%) and F6 still the lowest (~59.10%). At the final sampling point (90 min), nearly complete release was achieved by F5 (99.91%) and F3 (99.34%), confirming the efficiency of these formulations in promoting rapid and extensive drug release.

The observed variation in ACM release profiles among the sixteen formulations can be attributed to differences in the composition and ratios of oil and S_mix_. All ACM-SEME formulations demonstrated significantly higher release compared with the pure ACM suspension, confirming the advantage of the SEME in enhancing drug dissolution.

The early release patterns (at 5 and 15 min) suggest a burst release phenomenon, which reflects the efficient dispersion and emulsification of ACM upon contact with the dissolution medium. This can be explained by the small droplet size of ACM-SEME, which increases the surface area of drug exposure to the medium, thereby enhancing dissolution and release. In addition, the Smix components contribute to solubilization of ACM, further accelerating release.

The selection of the 90-min time point as a comparative benchmark in our SEME formulation optimization was guided by the observation that several formulations achieved near-complete drug release by this stage. This pragmatic choice aligns with established practices in formulation development, where plateaued release time points are routinely used for performance comparison. For example, a recent study on a solidified micelle system for atorvastatin adopted the time to reach 100% (or near-maximal) dissolution as the key parameter for evaluating release efficiency in their optimization design [58]. Similarly, methodologies utilizing D-optimal mixture designs for lipid-based microemulsion systems have employed early plateau points in release profiles to compare formulation performance across batches [59]. By using 90 min, we provide a consistent and robust reference for release efficiency that reflects real performance characteristics, enabling reliable formulation selection and advancement.

By evaluating the regression equation of the percentage of ACM released from ACM-SEME formulations (Table 2), it was discovered that the S_mix_ concentration has the most positive influence on drug release, with a huge coefficient of +260.59. This shows that raising S_mix_ considerably increases ACM release, most likely because it facilitates the development of smaller, more uniform droplets that increase the surface area for drug diffusion. S_mix_ also helps solubilize lipophilic drugs, enhancing their release into the aqueous medium. This effect was particularly evident in formulations such as F3 and F5, both of which had S_mix_ levels of 62% and exhibited superior ACM release of 99.34% and 99.91%, respectively. Water concentration also contributes positively to ACM release (+92.90), though to a lesser extent than S_mix_. Increased water content may enhance the diffusion gradient and facilitate drug transport out of the droplets. In addition, water helps maintain the fluidity of the continuous phase, which can accelerate release kinetics. Oil concentration, while still contributing positively (+76.53), has a less pronounced effect than Smix or water. A possible explanation is that although oil is essential for solubilizing lipophilic drugs, higher oil content may increase droplet size or viscosity, which can retard drug diffusion unless adequately balanced with surfactants. Moreover, higher oil content may lead to drug retention within the lipid phase, further reducing the amount of drug available for release into the aqueous medium [41,60,61].

The contour and 3D surface plots (Figure 4D) showed that higher drug release values are associated with formulations containing lower oil content and higher water levels. This pattern suggests that reducing the proportion of the oily phase enhances drug diffusion, likely due to a thinner interfacial layer and reduced viscosity as illustrated previously, which facilitates faster release into the surrounding medium [52,62]. Conversely, the regions with high oil content and low water levels correspond to significantly lower drug release values. This inverse relationship between oil content and release rate can be attributed to the oil-rich environment acting as a diffusion barrier, slowing the transport of the drug from the internal phase into the aqueous medium [63].

For optimization, the optimized formulation was selected based on the lowest PS, low PDI value, high ACM content, and high cumulative ACM percentage released after 90 min. Based on optimization obtained using design of experiment software, the F5, that contains 10% *w*/*w* oil, 62% *w*/*w* Smix, and 28% *w*/*w* water, was chosen as the optimized formulation with a desirability of 0.981.

### 3.8. Percentage Transmittance (T%) and Zeta Potential

The results of Percentage Transmittance (Table 1) showed a high level of clarity and consistency across all samples, with values ranging only between 99.31% and 99.77%. Such high transmittance indicates optically clear or almost transparent systems, implying successful SEME production. These findings demonstrate that the formulations accomplished effective self-emulsification, resulting in nano-sized droplets that scatter little light, improving optical clarity and formulation stability [64]. The result of zeta potential for optimized ACM-SEME optimized formulation was −26.98 ± 4.5 mV, indicating stability of the formulation.

### 3.9. FTIR Spectral Results and Interpretation

**Figure 6:**
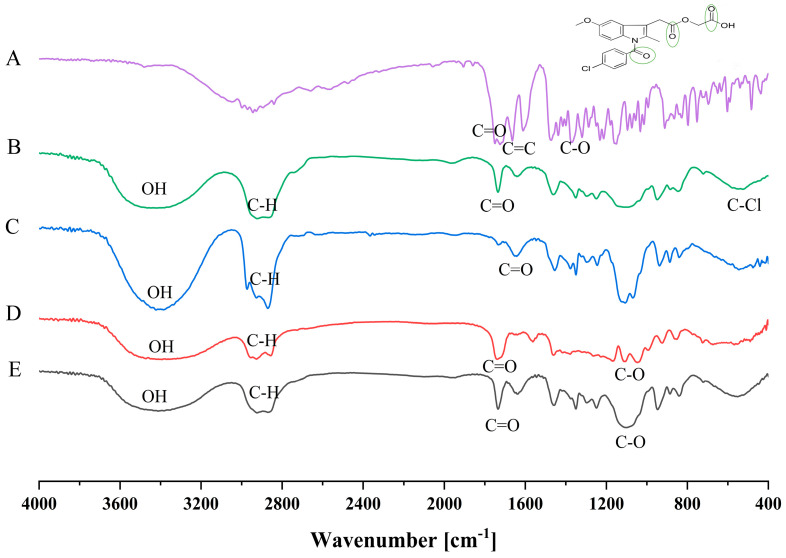
FTIR spectrums of A; ACM, B; Tween, C; Transcutol-P, D; Imwitor 308, E: ACM-SEME optimized formulation.

The FTIR spectra of ACM, the individual excipients, and the optimized ACM-SEME formulation are shown in Figure 5. The spectrum of pure ACM (Figure 6A) displayed distinct absorption bands consistent with its chemical structure. A broad O–H stretching vibration appeared between 2841 and 3047 cm^−1^, confirming the presence of hydroxyl groups. Strong carbonyl (C=O) stretching vibrations were observed at 1749 and 1724 cm^−1^, while a peak at 1610 cm^−1^ corresponded to aromatic C=C stretching. Additional bands at 1288, 1235, and 1213 cm^−1^ were attributed to C–O stretching of ester and ether linkages, and the peak near 540 cm^−1^ indicated C–Cl stretching from the chlorinated aromatic ring [4,65,66].

The FTIR spectra of the individual excipients showed their own characteristic peaks. Tween 20 (Figure 6B) exhibited a broad O–H stretching band around 3410 cm^−1^, aliphatic C–H stretching at 2924 and 2868 cm^−1^, and a sharp ester carbonyl peak at 1735 cm^−1^ [67]. Transcutol-P (Figure 6C) showed a broad O–H band at 3385 cm^−1^, aliphatic C–H bands at 2927 and 2870 cm^−1^, and a strong C–O stretching band at 1107 cm^−1^, consistent with its diethylene glycol monoethyl ether backbone. Imwitor 308 (Figure 6D) was characterized by a carbonyl ester peak at 1739 cm^−1^, O–H stretching at 3392 cm^−1^, C–H stretching at 2926 and 2856 cm^−1^, and C–O stretching between 1000 and 1200 cm^−1^, representing ester linkages that contribute to Imwitor’s emulsifying ability.

In the optimized ACM-SEME formulation (Figure 6E), notable spectral changes were observed compared with pure ACM. The broad O–H stretching band (2841–3047 cm^−1^), prominent in ACM, was absent in the SEME spectrum, suggesting hydrogen bonding or other intermolecular interactions between ACM and the hydrophilic groups of the excipients. Furthermore, the strong ACM carbonyl stretching peaks (1749 and 1724 cm^−1^) were diminished in intensity and slightly shifted, indicating involvement of the C=O groups in drug–excipient interactions. The attenuation and disappearance of these bands are consistent with hydrogen bonding or van der Waals forces between ACM and the hydrophilic domains of Tween, Transcutol, and Imwitor.

Importantly, no new peaks were detected in the ACM-SEME spectrum, confirming that these interactions are physical rather than chemical. There is no evidence of covalent bond formation, degradation products, or new functional groups. Thus, the incorporation of ACM into SEME occurs via noncovalent interactions (hydrogen bonding, van der Waals forces) that stabilize ACM in the formulation. This finding indicates that ACM’s chemical integrity was preserved, while its crystalline arrangement was disrupted upon incorporation into the SEME. The reduction of distinct ACM carbonyl and hydroxyl peaks suggests that the drug is no longer present in its native crystalline form but is instead molecularly dispersed within the excipient matrix in a partially amorphous state. Such molecular dispersion reduces lattice energy barriers and enhances drug wettability, leading to improved solubility and dissolution behavior. This is consistent with reports that lipid-based self-emulsifying systems stabilize poorly soluble drugs through hydrogen bonding and entrapment within amphiphilic domains, thereby preventing recrystallization during storage or upon dispersion. The FTIR findings confirm that ACM was successfully incorporated into the SEME system and stabilized via physical interactions with excipients, which play a role in the improved solubilization of ACM, which in turn is expected to enhance its oral bioavailability.

### 3.10. XRD and DSC

**Figure 7:**
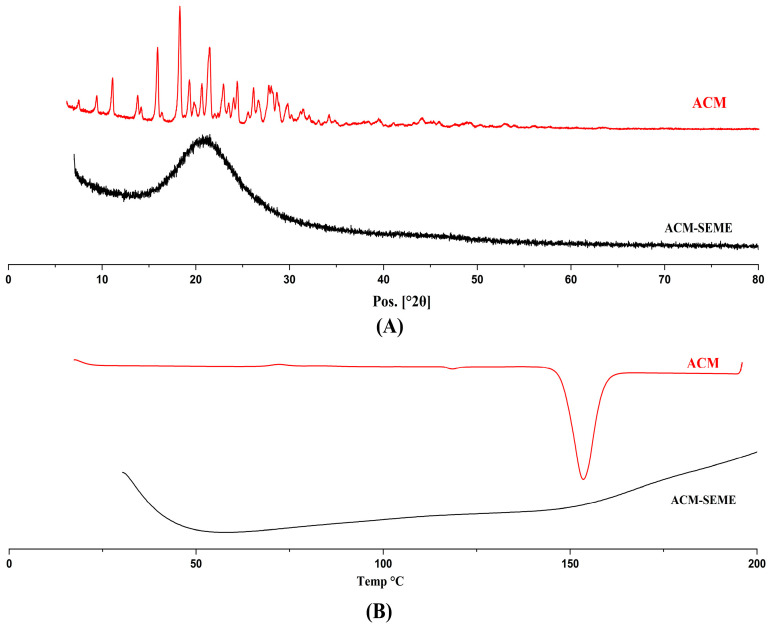
The XRD (**A**) and DSC (**B**) of ACM and ACM-SEME optimized formulation.

The results obtained from the XRD pattern of the ACM and ACM-SEME optimized formulation (Figure 7A) exhibited a complete absence of the characteristic crystalline peaks observed in the pure ACM. This transformation indicates a shift towards an amorphous or molecularly dispersed state of ACM within the ACM-SEME system. The disappearance of sharp diffraction peaks and the emergence of a diffuse halo pattern are hallmark features of amorphous materials, suggesting that the drug was successfully solubilized or molecularly dispersed within the formulation matrix. The amorphous nature of the ACM-SEME formulation observed in the XRD pattern can be explained by the molecular dispersion of ACM within the lipid-surfactant matrix during preparation. When ACM is first dissolved in oil and subsequently spray-dried with surfactants and carriers, the drug does not recrystallize upon solvent removal. Instead, it remains entrapped in a disordered, non-crystalline state within the solidified matrix. This behavior is well documented for lipid-based and self-emulsifying systems, where the rapid solidification process and strong intermolecular interactions between drug and excipients inhibit nucleation and crystal growth, thereby stabilizing the amorphous form [57,68,69]. This amorphization is expected to enhance dissolution behavior and potentially improve the oral bioavailability of ACM by circumventing the solubility limitations associated with its crystalline form. The results of XRD are in line with that obtained from DSC.

The DSC of pure ACM (Figure 7B) showed a strong endothermic peak at 156 °C corresponding to the melting temperature of crystalline ACM, suggesting its high crystalline nature. The DSC thermogram of the optimized ACM-SEME formulation (Figure 7B) reveals a broad endothermic peak, which may correspond to phase transition of the SEME components. The absence of the sharp peak at 156 °C in ACM-SEME thermogram and the appearance of a broad peak from 50 to 150 indicate that the ACM was changed from crystalline to amorphous, which will enhance the solubility of ACM. The interpretation, supported by the absence of crystalline reflections in DSC, indicates that although the final SEME powder appears solid after spray-drying, ACM does not exist as crystalline particles. Instead, it remains solubilized within the excipients and is entrapped in an amorphous solid dispersion rather than recrystallizing into its native crystalline form [70]. Similar behavior has been observed in other lipid-based and spray-dried systems, where strong drug–excipient interactions and rapid drying prevent recrystallization [71,72]

### 3.11. Morphological Analysis of Dried ACM-SEME Optimized Formulation

**Figure 8:**
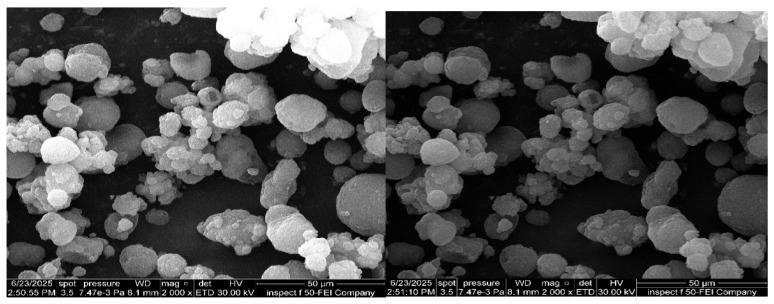
FESEM of ACM-SEME Optimized Formulation.

The results of FESEM obtained showed mono-spherical droplets, as illustrated in Figure 8, indicating the nanoscale of the obtained ACM-SEME.

### 3.12. In Vitro Release Study of Eudragit L100-Coated Capsules Filled with Spray-Dried ACM-SEME

The in vitro release of Eudragit L100-coated capsules containing spray-dried ACM-SEME revealed effective pH-dependent release behavior, which is compatible with Eudragit L100’s enteric qualities. At pH 1.2, which simulates the acidic environment of the stomach, no ACM release was observed over the 2-h release period, and the capsules remained intact. This confirms the acid resistance of the Eudragit L100 coating, thereby protecting the encapsulated ACM-SEME from potential degradation in the gastric environment. When the pH was adjusted to 6.8, imitating the intestinal environment, capsules disintegrated rapidly within 8 min, followed by complete ACM release within 2 min. This fast shift supports Eudragit L100 as a pH-sensitive polymer that dissolves above pH 6, ensuring targeted medication administration to the intestine [73,74]. The prompt and complete release of ACM also suggests that the spray-dried ACM-SEME formulation readily disperses and dissolves upon exposure to intestinal pH, which may enhance the drug’s bioavailability and therapeutic effectiveness.

### 3.13. Effect of ACM on the Noxious Time Threshold on Rats

**Figure 9:**
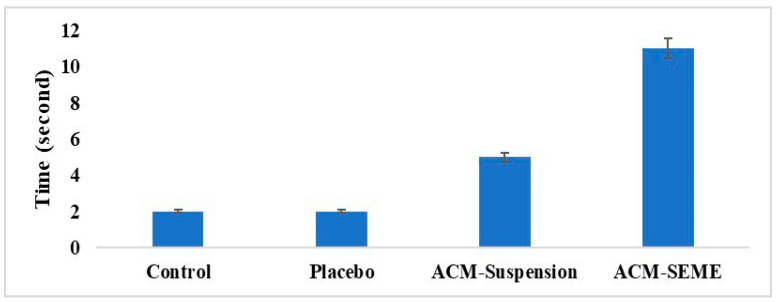
Effect of ACM on the Noxious Time Threshold in Rats.

In addition to formulation optimization, it is critical to assess the pharmacodynamic performance of the designed drug delivery system. Among the several experimental models used to measure analgesic efficacy, Eddy’s hot plate test is a well-known approach for assessing central antinociceptive activity in rodents. This approach evaluates the latency of reaction to a heat stimulus and so provides a valid indication of the analgesic potential of a test drug or formulation [27,75]. The data (Figure 9) showed that the ACM-SEME formulation significantly improves the analgesic efficacy of ACM, as evidenced by a longer latency to pain response in the hot plate test. This prolonged response time suggests better therapeutic efficacy, which is most likely related to the SEME system’s facilitation of drug solubilization, absorption, and subsequent bioavailability. The nano-sized droplets and S_mix_-assisted emulsification enable faster and more efficient drug penetration across gastrointestinal membranes, resulting in increased systemic exposure [27]. In contrast, the ACM suspension showed a small rise in pain threshold, indicating that drug absorption was limited due to ACM’s poor water solubility and slow onset of action. The control and placebo groups had the smallest delay times, showing the lack of intrinsic analgesic action in the vehicle and verifying the assay’s sensitivity. These findings align with previous reports highlighting the ability of SEME to improve the onset and magnitude of pharmacological responses, particularly for poorly water-soluble drugs. Therefore, the enhanced thermal pain tolerance in the ACM-SEME group underscores the system’s potential as an effective strategy for delivering NSAIDs with improved therapeutic outcomes [76,77].

### 3.14. Ex Vivo Permeation Study

The non-everted intestinal sac method, a common and well-established approach for assessing drug formulations’ intestinal absorption capability, was used in the ex vivo permeation investigation. Drug detection in receptor fluid indicates trans-epithelial penetration, which correlates to absorption into intestinal capillaries in vivo. The non-everted sac method has been successfully used in drug permeability tests and proven to correlate well with in vivo absorption, with various advantages over the everted sac, including enhanced tissue integrity and a simpler setup [78,79].

**Figure 10:**
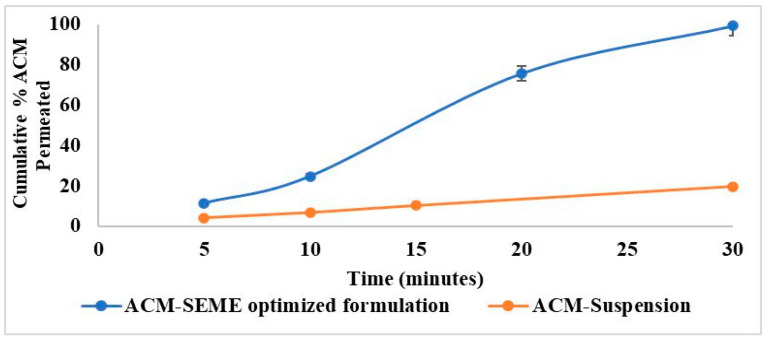
Ex Vivo Permeation of ACM from ACM Suspension and ACM-SEME Optimized Formulation.

The ex vivo permeation of the ACM profile of the optimized ACM-SEME formulation was compared to that of a conventional ACM suspension over a 30-min period (Figure 10). The ex vivo permeation study revealed a markedly superior dissolution and permeation performance of the optimized ACM-SEME formulation in comparison to the ACM suspension. At 5 min, the cumulative percentage release from the ACM-SEME was approximately 11.53%, whereas the suspension was released only around 4.26%. This trend continued, with the ACM-SEME reaching about 25% in 10 min, 75% in 20 min, and nearly 99.5% release at the end of 30 min. This enhancement can be attributed to the features of the microemulsion system. First, the large surface area of the nano-size droplets facilitates faster drug diffusion into the dissolution medium. Additionally, the presence of S_mix_ in the SEME aids in solubilizing ACM, thereby enhancing its apparent solubility and release rate. S_mix_ also facilitates drug permeation by disrupting the intestinal epithelium through membrane fluidization, drug solubilization, and partial lipid extraction. Moreover, the micellar structures that form during emulsification can further improve drug partitioning into the aqueous phase [27,80]. In contrast, the suspension displayed a much slower release profile, reaching only ~20% in 30 min, likely due to the poor wettability, larger PS, and inadequate solubilization of the crystalline drug particles. The rapid and almost complete release observed from the ACM-SEME within 30 min underscores the system’s ability to bypass the dissolution step, a major limitation in the bioavailability of BCS Class II drugs like ACM.

The release of ACM was found to be pH-dependent, with a significantly higher release observed at pH 7.4 compared to pH 6.8, This behavior is attributed to the increased ionization and solubility of acemetacin at neutral pH, which enhances its dissolution and diffusion. The faster permeation observed in the ex vivo intestinal model, compared to the in vitro release assay, can be explained by differences in formulation state and experimental barriers. In vitro release, using liquid SEME across a dialysis membrane, depends on diffusion across both the oil–water interface and the membrane, resulting in a slower profile (up to ~90 min) which further slowed by formulation viscosity. In contrast, the ex vivo permeation used spray-dried SEME, which upon reconstitution disperses into finer droplets with ACM in a partially amorphous, high-energy state. This generates a steep concentration gradient and enables more rapid permeation within 30 min. Moreover, the non-everted intestinal sac provides biological absorption pathways (passive transcellular, paracellular, and lipid-affinity transport) that significantly enhance drug passage compared to the purely diffusional dialysis model. This phenomenon is consistent with recent studies wherein spray-dried lipid nano dispersions, upon reconstitution, maintained droplet integrity with only slight increases in size and exhibited significantly quicker dissolution profiles than liquid forms [81]. Studies such as one examining praziquantel-loaded self-emulsifying systems confirm that SEME can achieve up to 18-fold increases in apparent permeability in ex vivo models versus powder forms [82]. The convergence of the in vitro and ex vivo data confirms that the SEME system ensures efficient drug release and promotes effective intestinal permeation of ACM, underscoring its potential to enhance oral bioavailability.

## 4. Conclusions

In the current work, SEME was successfully developed and optimized to improve the solubility, dissolution rate, and oral bioavailability of ACM. The experimental design analysis revealed that F5 was the optimal formulation, with a desirable small PS, low PDI, high ACM content, and high cumulative percentage of ACM released after 90 min.

The dried ACM-SEME retained structural integrity and drug compatibility, as evidenced by FTIR, XRD, DSC, and FESEM. Encapsulating the dry ACM-SEME in enteric-coated capsules provided gastrointestinal protection and pH-dependent drug release supporting the enteric coating’s efficiency. In vivo testing with Eddy’s hot plate test indicated a substantial rise in pain threshold, demonstrating improved antinociceptive efficacy of the adjusted formulation. Furthermore, ex vivo permeation experiments demonstrated increased drug permeability across intestinal membranes. Overall, the ACM-SEME formulation is a promising strategy for oral ACM delivery, combining enhanced solubility and bioavailability with fewer gastrointestinal adverse effects. This platform could be extended to include more BCS Class II drugs with comparable solubility issues.

## Figures and Tables

**Table 1:** The dependent and independent variables for the prepared ACM-SEME using D-optimal design.

Formulation	Imwitor 308 Oil % *w*/*w*	S_mix_ % *w*/*w*	Water % *w*/*w*	PS (nm)	PDI	ACM Content %	% Released After 90 min	T%
F1	25	65	10	40.03 ± 2.04	0.29 ± 0.01	96.56 ± 0.57	81.63 ± 0.25	99.31 ± 0.24
F2	18	59	22	34.09 ± 1.34	0.2 ± 0.02	99.49 ± 0.59	84.39 ± 0.55	99.54 ± 0.11
F3 *	10	62	28	20.21 ± 0.58	0.16 ± 0.01	99.61 ± 0.23	99.349 ± 0.54	99.77 ± 0.35
F4	15	59	27	32.85 ± 0.98	0.23 ± 0.03	99.4 ± 0.14	85.03 ± 0.67	99.77 ± 0.21
F5 *	10	62	28	21.26 ± 0.74	0.18 ± 0.02	99.75 ± 0.14	99.91 ± 0.24	99.77 ± 0.15
F6	25	55	20	41.95 ± 1.25	0.32 ± 0.01	98.21 ± 0.16	80.57 ± 0.65	99.77 ± 0.12
F7	25	55	20	42.36 ± 1.24	0.31 ± 0.03	98.54 ± 0.18	79.71 ± 0.32	99.54 ± 0.14
F8	19	55	26	37.75 ± 1.32	0.18 ± 0.02	98.64 ± 0.54	83.97 ± 0.78	99.77 ± 0.18
F9	19	65	16	35.5 ± 0.85	0.16 ± 0.01	98.31 ± 0.65	84.18 ± 0.57	99.77 ± 0.16
F10	10	55	35	30.01 ± 0.75	0.22 ± 0.02	98.31 ± 0.33	93.53 ± 0.86	99.54 ± 0.36
F11	25	65	10	43.25 ± 1.23	0.33 ± 0.01	96.9 ± 0.25	81.63 ± 0.63	99.54 ± 0.24
F12	14	65	21	30.69 ± 0.89	0.27 ± 0.03	98.09 ± 0.65	90.34 ± 0.98	99.54 ± 0.19
F13	14	65	21	30.65 ± 1.01	0.28 ± 0.02	98.21 ± 0.46	89.92 ± 0.46	99.77 ± 0.32
F14	25	60	15	39.35 ± 1.36	0.31 ± 0.2	98.31 ± 0.47	81.208 ± 0.78	99.77 ± 0.24
F15	14	55	31	31.39 ± 0.87	0.19 ± 0.1	98.72 ± 0.87	87.79 ± 0.96	99.31 ± 0.34
F16	10	55	35	30.09 ± 0.57	0.23 ± 0.01	98.09 ± 0.45	92.47 ± 0.85	99.77 ± 0.25

S_mix_ (Tween 20 and Transcutol-P in a 3:1 ratio), * ACM-SEME optimized formulation, PS (globule size), PDI (polydispersity index), and T% (Percentage Transmittance).

**Table 2:** The Fit Statistics for the dependent variable.

	PS	PDI	ACM Content	% Released
Model	Special Quartic	Cubic	Cubic	Special Quartic
*p*-value	<0.0001	0.0003	<0.0001	<0.0001
F-value	56.26	29.18	46.71	208.16
R^2^	0.9847	0.9777	0.9859	0.9958
Adjusted R^2^	0.9672	0.9442	0.9648	0.9910
Predicted R^2^	0.8629	0.8260	0.8797	0.9212
Adeq Precision	23.0750	13.6299	22.7263	42.5470
The coded equation for the dependent variable				
Response	Equation			
PS	PS = +56.42 A − 31.41 B + 29.87 C + 84.88 AB − 13.74 AC + 36.99 BC − 699.26 A^2^BC + 985.06 AB^2^C + 190.48 ABC^2^			
PDI	PDI = +1.21 A + 24.90 B + 0.2249 C − 48.69 AB − 1.94 AC − 44.90 BC + 49.94 ABC + 27.21 AB(A-B) − 0.7593 AC(A-C) − 23.50 BC(B-C)			
ACM content	ACM content = 99.3596 A − 86.5521 B + 98.1989 C + 340.7 AB − 1.17886 AC + 333.557 BC − 316.671 ABC − 209.148 AB(A-B) − 5.06269 AC(A-C) + 158.716 BC(B-C)			
Cumulative percentage released	Cumulative percentage released = +76.53 A + 260.59 B + 92.90 C − 285.58 AB − 12.80 AC − 193.84 BC + 1172.86 A^2^BC − 1740.66 AB^2^C − 5.77 ABC^2^			

Where A is oil percentage, B is Smix percentage, and C is water percentage.

## Data Availability

The raw data supporting the conclusions of this article will be available by the authors on request.

## Abbreviations

The following abbreviations are used in this manuscript:

ACM	Acemetacin
SEME	Self-Emulsifying Microemulsion
UV	Ultraviolet
NSAID	Non-steroidal anti-inflammatory drug
COX	Cyclooxygenase
PEG400	Polyethylene Glycol 400
PEG600	Polyethylene Glycol 600
PS	Particle size
PDI	Polydispersity Index
FTIR	Fourier Transform Infrared Spectroscopy
FESEM	Field Emission Scanning Electron Microscopy
XRD	X-ray diffraction
DSC	Differential Scanning calorimetry
3D	Three-Dimensional
SD	Standard Deviation
PBS	Phosphate-Buffered saline
USP	United States pharmacopeia
